# Gender Differences in Social Support Received by Informal Caregivers: A Personal Network Analysis Approach

**DOI:** 10.3390/ijerph16010091

**Published:** 2018-12-31

**Authors:** María Nieves Rodríguez-Madrid, María del Río-Lozano, Rosario Fernandez-Peña, Jaime Jiménez-Pernett, Leticia García-Mochón, Amparo Lupiañez-Castillo, María del Mar García-Calvente

**Affiliations:** 1 Fundación para la Investigación Biosanitaria de Andalucía Oriental (FIBAO), 18012 Granada, Spain; nieves.rodriguez.ext@juntadeandalucia.es; 2 Andalusian School of Public Health, Consejería de Salud, Junta de Andalucía, 18080 Granada, Spain; jaime.jimenez.easp@juntadeandalucia.es (J.J.-P.); leticia.garcia.easp@juntadeandalucia.es (L.G.-M.); amparo.lupianez.easp@juntadeandalucia.es (A.L.-C.); mariadelmar.garcia.easp@juntadeandalucia.es (M.d.M.G.-C.); 3 Biomedical Research Centre (ibs.GRANADA), 18080 Granada, Spain; 4 Department of Nursing, SALBIS Research Group, Nursing Research Group IDIVAL, University of Cantabria, 39008 Santander, Spain; roser.fernandez@unican.es; 5 Public Health Research Institute (IRSPUM), University of Montreal, Montreal, QC H3N 1X7, Canada; 6 CIBER en Epidemiologia y Salud Pública (CIBERESP), 28029 Madrid, Spain

**Keywords:** social network analysis, personal network analysis, social support, informal care, caregivers, gender differences

## Abstract

Social support is an important predictor of the health of a population. Few studies have analyzed the influence of caregivers’ personal networks from a gender perspective. The aim of this study was to analyze the composition, structure, and function of informal caregiver support networks and to examine gender differences. It also aimed to explore the association between different network characteristics and self-perceived health among caregivers. We performed a social network analysis study using a convenience sample of 25 female and 25 male caregivers. A descriptive analysis of the caregivers and bivariate analyses for associations with self-perceived health were performed. The structural metrics analyzed were density; degree centrality mean; betweenness centrality mean; and number of cliques, components, and isolates. The variability observed in the structure of the networks was not explained by gender. Some significant differences between men and women were observed for network composition and function. Women received help mainly from women with a similar profile to them. Men’s networks were broader and more diverse and they had more help from outside family circles, although these outcomes were not statistically significant. Our results indicate the need to develop strategies that do not reinforce traditional gender roles, but rather encourage a greater sharing of responsibility among all parties.

## 1. Introduction

The number of dependent people in Spain is rising, partly because of the progressive aging of the population. Most people who require assistance for activities of daily living are cared for by informal caregivers, who do not receive payment for this work and are largely members of the family [1]. 

Informal caregiving is unpaid work generally provided by a person (frequently a woman) who is emotionally attached to the person in need of care; it is a private, domestic (invisible) task that is undervalued both socially and economically [2]. Almost three-quarters (73.8%) of informal caregivers in Spain are women, and the percentage is even higher, at 77.5%, in Andalusia, southern Spain [3]. These gender differences are also evident in the type of care provided. Recent findings indicate that women devote more time to caregiving, receive less support, and perform more tasks that are potentially detrimental to their health [4,5].

Numerous studies have found that caregiving has both negative physical and psychological health effects [6,7,8,9], and the factors that modulate these effects include level of dependency and the type and intensity of care required. Compared with non-caregivers, caregivers have been found to have worse health-related quality of life, greater physical and mental health problems, and less healthy life habits [6,8,9]. These effects also vary from individual to individual, and those who experience the greatest deterioration in physical and mental health are women; older caregivers; and caregivers from a low socioeconomic background, with poor previous health, and with little social support [9]. 

Caregiving also has a different effect on men and women [4]. According to the most recent survey conducted on disability, personal autonomy, and dependency in Spain [4], 40% of female caregivers reported impaired health (vs. 24% of men) and 35% felt depressed (vs. 20% of men). Female caregivers also experience greater mental illness and worse self-perceived health [10,11]. The literature is inconclusive, however, on how men and women differ in terms of burden experienced [12]. While some studies indicate that women are more severely burdened as a result of their greater caregiving role [10], others suggest that men may perceive a heavier burden because they have to fulfil a role for which they have not been socially prepared [13]. 

Social support is one of the main predictors of caregiver health [14,15,16], and it is particularly important for relieving the burden as it gives caregivers a greater sense of control over their lives [17]. According to a recent study by our group [18], women seek less help than men and rely more on support from relatives than from formal support systems or paid help. These and similar results on social support structures and functions call for a more in-depth investigation of caregivers’ personal support networks. One interesting theoretical and methodological approach for investigating such networks is social network analysis (SNA) [19,20], which is a useful tool for shedding light on how different patterns of social support affect caregiver health [21]. There are two traditional approaches in SNA: the sociocentric approach, which looks at the whole network, and the egocentric approach, which looks at a focal actor, known as the “ego”. Studies of sociocentric networks focus on ties within a socially defined group. Egocentric studies, however, look at the ties between an individual (the ego) with different people (known as “alters”) in their different social structures (relatives, work colleagues, neighbors, etc.). These networks of people are known as “egocentric networks” [22,23]. Moreover, personal networks are a subset of the more ample concept of egocentric networks. This analysis is typically reserved for those studies where researchers focus on ensembles of social relationships surrounding an ego, in all social settings in which ego is embedded. [23] This is the approach of our study. 

It has been shown that the structure and cohesion of personal networks are important factors for social support exchanges and that support is key for resolving personal concerns and health problems [21]. Nonetheless, very few studies have analyzed the association between social support and caregiver health from an SNA approach and even fewer have taken a gender approach. 

One SNA study helped to characterize the social support networks of people with chronic pain and highlighted the important roles that network structure, composition, and function have in terms of the provision of support and satisfaction with support received [24]. Another study used a similar approach to analyze the personal networks of caregivers of people with Alzheimer’s disease and found that smaller networks were associated with greater burden [25]. The study also showed that people’s networks were mostly comprised of women who lived in the same city and offered emotional but little material support. Studies such as these invite further investigation of personal networks and support systems used by both male and female caregivers as a preliminary step towards mitigating gender inequalities in the distribution of care and understanding the impact on caregiver health. 

The main aims of the present study were to characterize the structure, composition, and function of personal caregiver networks and to investigate whether these characteristics vary between women and men. To do this, we examined differences between male and female networks in terms of the sociodemographic profile of the members, the structure, the type of support received, and the composition in terms of the characteristics of people providing support in the form of help with specific caregiving tasks. As a secondary objective, we also explored the association between the characteristics of the networks and self-perceived caregiver health.

## 2. Materials and Methods

### 2.1. Study Design

We undertook a cross-sectional SNA study [19], in which we analyzed the personal networks of a convenience sample of informal caregivers in Granada, Spain. This descriptive study is the first of several quantitative and qualitative studies that will be produced within the framework of a larger study based on information collected in the interviews conducted for this study. 

The study population was formed by informal caregivers aged 18 years or older living in a family home in the province of Granada, Spain who provided unpaid care to a dependent person living in the same or another home. 

Participants had to form part of the CUIDAR-SE project, which is a longitudinal study being conducted by our group that involves 404 people registered as caregivers in the Granada Health District. Already having detailed information on the profile of these caregivers, on the care they provide, and on their health is a particular strength of this study. Participants were included in a registry of caregivers from the primary health care services. All the caregivers were taking care of a person in a situation of dependency that required help to perform the activities of daily life for at least one year. This dependence could be caused by very diverse causes, physical or mental or both. We excluded people who might have had difficulty answering the survey questions because of language barriers or circumstances that would have prevented them from participating in the follow-up stages.

We selected a convenience sample of 50 caregivers, stratified by gender (25 women and 25 men). Other criteria analyzed to investigate the heterogeneity of each of the gender groups were age, level of education, time as caregiver, place of residence (urban vs. rural), and level of burden according to the Zarit Burden Interview (with a total score ranging from 22 to 110, where ≤46 corresponds to no burden, 47–55 to mild burden, and ≥56 to intense burden) [26].

These variables and self-perceived health were collected through the structured questionnaire of the CUIDAR-SE project. The question about self-perceived health was asked as follows: “In general, you would say that your health is excellent, very good, good, fair, or bad (only one option)”. The first three categories were grouped in “good health” and the last two in “poor health”.

### 2.2. Data Collection

The information of networks was collected during personal interviews held between September 2017 and March 2018. The people selected to participate in the survey were contacted by telephone. The interviews were held in a place chosen by the interviewee and all efforts were made to create an intimate environment. Prior to the interview, the participants were informed of the objectives and ethical issues surrounding the project and asked to sign an informed consent form agreeing to the interview and its tape-recording. 

The information was recorded using the EgoNet software program [27,28] via an ad hoc structured questionnaire designed by the research team; the questionnaire was previously piloted among five caregivers. An English translation of the original Spanish questionnaire is attached in the Appendix A. Using the EgoNet name generator module, the interviewer asked the interviewees to name 25 people from their different social spheres who “form part of their lives”. The interviews lasted between 60 and 75 min and were tape-recorded. 

### 2.3. Data Analysis

EgoNet provided the numbers and percentages for the different variables describing the composition and function of each of the personal networks analyzed, the means of the structure variables, and the centrality metrics for each of the alters in the networks. It also produced graphs depicting some of the most representative results related to the composition of the male and female caregivers’ networks.

A descriptive analysis stratified by gender was made in the statistical program SPSS. We have taken into account the following dimensions and variables of analysis in order to characterize the structure, composition, and function of personal networks of the caregivers: (a) composition of personal caregiver networks: gender, age, place of residence, type of relationship, and proximity; (b) structure of the personal networks: density, degree centrality mean, betweenness centrality mean, number of isolates, number of components, and number of cliques; and (c) functional social support: type of help with specific care tasks, financial support, and emotional support. For the descriptive analysis, we analyzed frequencies and percentages. Associations with gender were investigated using the *t* test for structural variables and the χ^2^ test for composition and function variables. *Density* refers to the number of ties as a percentage of all possible ties in the network; *degree centrality* refers to the total number of alters to which each alter is directly connected; *betweenness centrality* refers to the extent to which an alter lies on the shortest paths between other alters; *isolates* are alters who are connected to the ego, but not to anybody else; *components* are a set of alters who are connected to one another directly or indirectly; and *cliques* are a set of alters who are all directly tied to each other [20,29]. 

We performed three bivariate analyses via binary logistic regression to examine associations between the dependent variable—self-perceived health (good vs. bad)—based on information collected in the CUIDAR-SE project, and three independent variables related to the characteristics of the personal networks: (a) degree centrality mean (structure); (b) type of relationship, that is, presence of direct relatives (composition); and (c) help with specific caregiving tasks (function). The strength of association was estimated using odds ratios with 95% confidence intervals. All the analyses were stratified by age, but not by gender, as gender was not found to be significantly associated with self-perceived health.

## 3. Results

### 3.1. Characteristics of the Sample

The sample was formed by 25 men and 25 women. Although multiple variables were analyzed within each gender group to investigate sources of heterogeneity (age, level of education, place of residence, time providing care, and caregiving burden), the results showed only slight differences between the groups. The main ones are summarized in Table 1. 

Men were older than women; 52% were aged over 65 years versus 28% of women. Women were less educated, as 60% (vs. 52% of men) had only secondary education or less. A slightly higher proportion of women lived in a rural environment (52% vs. 48% for those living in an urban environment). Men were more likely to live in a town or city (56% vs. 44%). Almost a third of both men and women had been providing care for less than two years and around half had been doing it for between 2 and 10 years. Just 20% of men and 16% of women had been working as informal caregivers for over 10 years. A majority of both men and women were severely burdened (56%) and 40% of women and 36% of men reported good health. 

### 3.2. Network Composition

The main differences between the composition of women’s and men’s personal networks are shown in Table 2. Women were predominant in both networks and comprised 64.3% of the alters in the women’s networks and 51.4% in the men’s. 

In total, 45.0% of network members were aged between 46 and 65 years; the distribution of men among the different age groups was more diverse. Women also had a higher number of alters living in the same house as men (5.1% vs. 2.9%). The alters in the men’s personal networks were mainly located in the same town or province (79.8%). In other words, men had more support from people living in the same province, but not so much from people living in the same household. 

There were also differences, albeit without statistical significance, between men and women in terms of the type and closeness of the relationship they had with their alters. Direct relatives (partners, children, parents, siblings, and others) and neighbors were more common in the women’s networks. Women also received less support from work colleagues and external support services (paid professional or non-professional help) than men. Men had a slightly lower proportion of geographically and affectively close contacts than women (82.7% vs. 79.8%).

**Figure 1:**
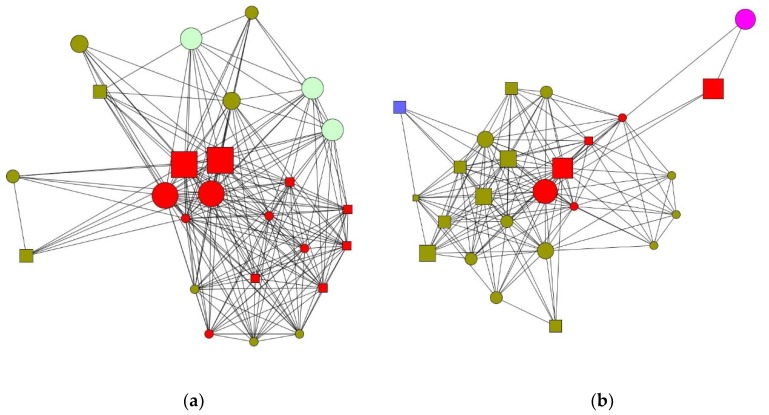
(**a**,**b**) Women are depicted as circles and men as squares. The size of the nodes shows place of residence, with a larger size depicting greater geographical proximity. Type of relationship is indicated by colors, with red indicating direct relatives; olive green, friends; light green, neighbors; blue, work colleagues; and pink, external help. Graphs depicting the personal networks of female and male caregivers.

The following graphs show differences in composition for a 48-year-old women (Figure 1a) and a 37-year-old man (Figure 1b). 

### 3.3. Network Structure

Table 3 summarizes the results for the structural variables analyzed in the different personal networks. Density of the personal network and degree centrality mean were slightly higher for men. The differences, however, were not statistically significant. The differences between women and men for mean betweenness and for numbers of components, cliques, and isolates were also not significant. The fact that the networks were cohesive, with few isolates, and structured around a single component, would explain this lack of structural variation.

### 3.4. Network Social Support Function

As can be seen in Table 4, approximately half of the network members offered emotional support, with a significant association between emotional support and gender. A lower proportion of alters provided financial support. 

More women than men provided help with specific caregiving tasks in both types of networks, but the difference was greater in the men’s networks. 

**Figure 2:**
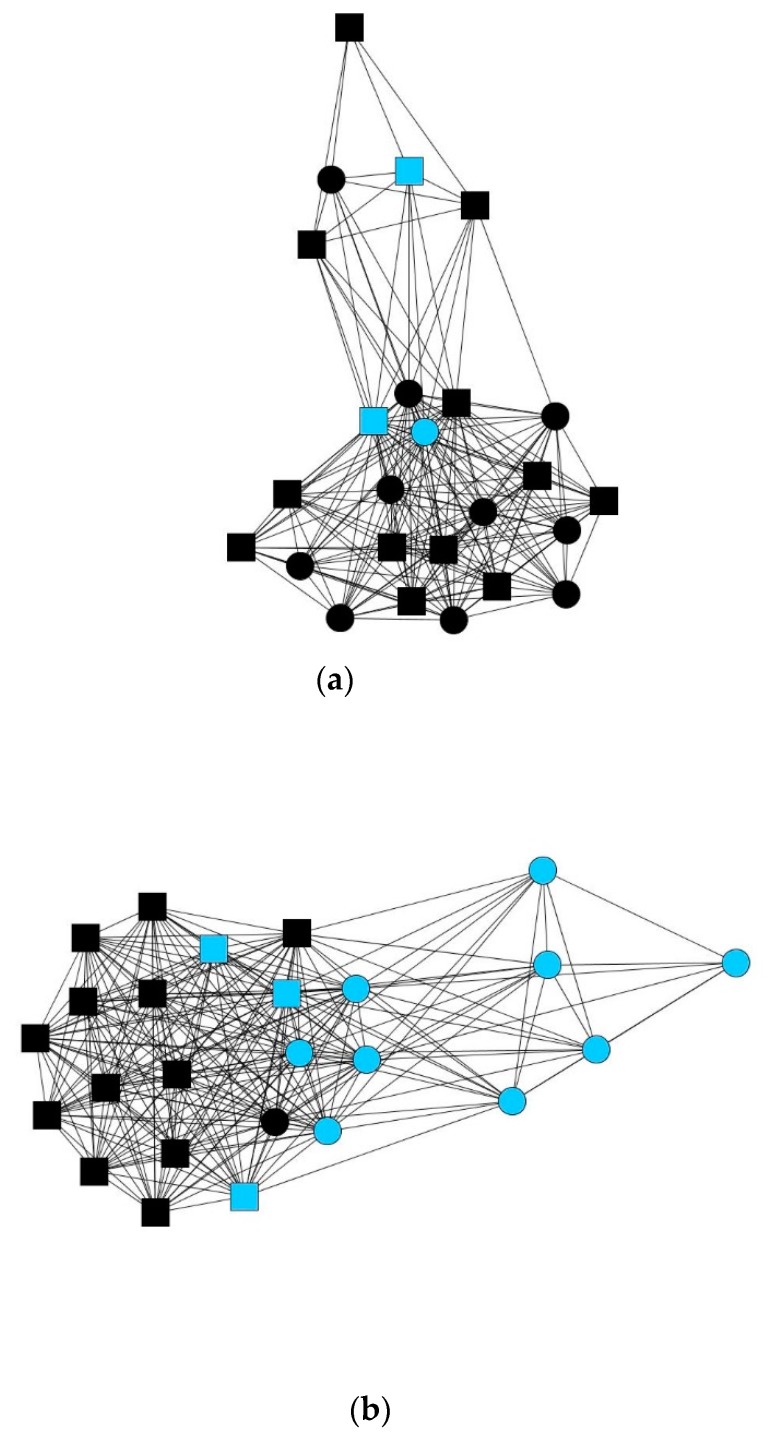
Female alters are shown as circles and male alters as squares. Blue indicates alters who provide help with specific caregiving tasks. Black indicates no help with specific tasks. Graphs depicting the personal networks of female (**a**) and male (**b**) caregivers.

The graphs in Figure 2 illustrate how women received less help with specific caregiving tasks (**a**) than men (**b**), who receive considerable support from women in this area. 

Men received more help than women with tasks such as personal care or household chores, which is where the greatest gender-based difference was noted (45.6% of men received help with household chores compared with 35.3% of women). Women, however, received more help than men with caregiving tasks outside the home and mobility tasks. 

Special mention should also be given to differences observed regarding the composition of alters providing help with specific caregiving tasks. Men received more support from people aged between 46 and 64, while women had more helpers in the younger age brackets and in the 65 and older bracket. Women also received more help from direct relatives and people living with them. Paid help, however, was less present in their networks. 

### 3.5. Associations between Network Characteristics and Self-Perceived Health

Women had a worse perception of their health than men, although the association between gender and self-perceived health was not significant. 

For the whole group combined and considering men and women in the same age groups, the likelihood of perceiving oneself to be in good health decreased as the number of direct relatives in the personal networks increased. This association, however, was not statistically significant.

To the contrary, the likelihood of perceiving poor health (again for men and women of a similar age) decreased with increasing degree centrality mean.

Finally, for men and women in the same age bracket, we observed a non-significant association between a higher number of alters and poor self-perceived health. Women had a worse perception of their health than men, although the association between gender and self-perceived health was not significant (Table 5).

## 4. Discussion

The results of this study add to the little information that has been published on the association between informal caregiving and health from the perspective of SNA and gender. Our characterization of support networks provides important insights that help to understand the different ways in which men and women manage their caregiving responsibilities and use resources from their personal network to meet these needs. We found some significant gender differences between the networks in terms of composition (gender, age, place of residence) and function (emotional support), but not in structure. A limitation of the study is the poor statistical significance observed, perhaps because of the small size of the sample. Nonetheless, some evidence in other studies could be useful for this discussion. 

Although the network structures varied somewhat, the differences between genders were not significant. Men and women did differ, however, in terms of their alters and the type of support received. Although gender differences in network composition have already been described [30], it is logical that the networks in our study are cohesive and have little structural diversity, as they include a group of people devoted to daily caregiving, and thus with limited opportunities to be active in other social groups or geographic areas.

The personal networks of both men and women in our study featured a high proportion of women, although more so in the latter case. Women’s personal networks are mainly composed of other, predominantly middle-aged, women who were either living in the same home or in another province. Men had almost an identical number of male and female alters in their networks and their ages were more diverse; men also received more help than women from colleagues and paid services. This predominance of women in the networks is consistent with findings from other studies. One study of caregivers of dependent elderly people concluded that caregivers with networks featuring more women received more help than those with networks with a predominance of men [31]. This supports evidence that men have broader and more diverse support networks and thus better access to social capital [32,33,34]. 

Findings from the literature also indicate that certain sociodemographic characteristics, such as gender, age, and social class, are frequently similar across networks, in line with the principle of homophily [35,36]. Nevertheless, the results of our study also show that relationships between caregivers and their support networks are determined more by specific support needs. These needs are seen as a priority by caregivers and their families. The predominance of middle-aged women among informal caregivers possibly indicates that both men and women choose a female caregiver, because this is what is socially and historically expected [37,38]. 

In our study, we have not observed statistically significant differences. However, our results could be consistent with those that have been found in other studies, that is, both men and women received more informal support from women than from men. Those providing help to men tended to be middle-aged, while those providing help to women tended to be in younger age brackets or 65 years or older. It is also noteworthy that compared with men, women use paid services less and received less support from people living close by. The profile of alters providing help with specific caregiving tasks coincides with reports in the literature. There is evidence that men play a complementary, secondary role in family caregiving and also use external services more [32,39,40,41]. Likewise, geographic distance has been found to be a limiting factor for the provision of help with specific caregiving tasks [31,33,42,43]. 

The literature suggests that men seem to find it easier to seek help from people within their close social circles, be they work colleagues, friends, or neighbors, and that they also see this help as legitimate considering they are fulfilling a role for which they have not prepared. Our results could be in line with this, although they are not significant. The situation of women, by contrast, might reflect a certain refusal on their part to seek help from friends or neighbors to fulfil a role for which they are supposedly prepared for, whatever the circumstance. These differences in help-seeking patterns support reports from other studies conducted in Spain [5]. Men’s greater ease in asking for help, combined with the more varied composition of their support personal networks, might also explain the slightly higher network density and degree centrality mean values observed for men. These results should, however, be interpreted with care, as other factors, such as time providing care and management of crisis periods, could be confounding effects that should be addressed in future studies.

The findings of our survey show that both men and women receive very little financial support. Emotional support was greater in both groups, supporting previous findings. One study of 23 caregiver networks found that 65.6% of alters offered emotional support compared with just 17.3% offering material support [25]. The SNA study of support received by caregivers of people with chronic pain showed a similar predominance of emotional support [24].

As for specific types of help with caregiving tasks, our findings suggest that men receive more assistance with personal care and less assistance with physical mobility tasks. Women, by contrast, receive more support for caregiving tasks outside the home. The men, and in particular the women interviewed, reported that they received little help with household chores. Although this does not have statistical significance, according to a recent study published by our group [18], women are more likely to look after nursing-type tasks (91%), while men take more responsibility for physical mobility tasks (89%). The study also highlighted how men and women used formal support services differently, with men making greater use of paid help and home and instrumental help (meals and transport), and women making greater use of state allowances and day and respite care centers. 

Our results did not show statistical significance in the relationship between care and self-perceived health. However, the evidence from other studies leads us to the need to deepen this relationship from complementary approaches, such as qualitative ones. In fact, there is clear evidence from other studies that women report worse health because of the greater burden they bear and that the differences between men and women in this respect shrink or disappear when the burden on men increases [44]. Observations such as these open the door for new lines of research. Having help from more direct relatives was associated with better self-perceived health, while more help with specific caregiving tasks was associated with a worse perception of health. Feeling emotionally supported and maintaining close ties with alters could be key factors for protecting the health of caregivers. These interpretations, however, are limited by the small sample and the cross-sectional nature of our study. Our observations must be followed up by more studies and in-depth analyses of the qualitative data contained within the narratives collected for this study. We hope that future findings will guide strategies that will help to set in action or strengthen different forms of support for informal caregivers from a perspective that addresses gender issues and promotes greater sharing of responsibilities among all the social agents involved. 

## 5. Conclusions

The findings of this study provide preliminary data for helping to understand the different ways in which male and female caregivers interact with members of their personal networks to meet their caregiving needs. The poor statistical significance of our study could be because of the small size of the sample. The personal networks of both genders were similar in structure, but varied significantly in terms of composition and function. Men had broader, more diverse networks than women and also received more support from alters outside the family circles, such as work colleagues and paid professionals and non-professionals. Women’s networks were less diverse and mainly featured women with similar sociodemographic profiles to theirs and often from the same family. Our findings highlight the need to develop public strategies that do not reinforce traditional gender roles, but rather encourage a sharing of responsibility among all stakeholders.

## Figures and Tables

**Table 1:** Characteristics of the male and female caregivers (egos).

Variables		Men	Women	Total
		*n* = 25	*n* = 25	*n* = 50
		*n* (%)	*n* (%)	*n* (%)
Age	<65 years	12 (48)	18 (72)	30 (60)
	≥65 years	13 (52)	7 (28)	20 (40)
Educational level	Secondary education or lower	13 (52)	15 (60)	28 (56)
	Higher education	12 (48)	10 (40)	22 (44)
Place of residence	Rural	11 (44)	13 (52)	24 (48)
	Urban	14 (56)	12 (48)	26 (52)
Time providing care	<2 years	8 (32)	8 (32)	16 (32)
	2–10 years	12 (48)	13 (52)	25 (50)
	>10 years	5 (20)	4 (16)	9 (18)
Burden	None	5 (20)	5 (20)	10 (20)
	Mild	6 (24)	6 (24)	12 (24)
	Severe	14 (56)	14 (56)	28 (56)
Self-perceived health	Good	9 (36)	10 (40)	19 (38)
	Poor	16 (64)	15 (60)	31 (62)

**Table 2:** Composition of male and female caregiver personal networks.

Characteristics of Alters		Male Egos	Female Egos	All Egos	*p*-Value
		*n* = 625 Alters	*n* = 625 Alters	*n* = 1250 Alters	
		*n* (%)	*n* (%)	*n* (%)	
Gender	Male	304 (48.6)	223 (35.7)	527 (42.2)	*p* < 0.001 *
	Female	321 (51.4)	402 (64.3)	723 (57.8)	
Age	<25 years	62 (9.9)	53 (8.5)	115 (9.2)	*p* = 0.008 *
	25–45 years	192 (30.7)	154 (24,6)	346 (27.7)	
	46–65 years	218 (34.9)	281 (45.0)	499 (39.9)	
	>65 years	139 (22.2)	124 (19.8)	263 (21.0)	
Place of residence	Ego do not know//No answer	14 (2.2)	13 (2.1)	27 (2.2)	*p* = 0.010 *
	Same home as caregiver	18 (2.9)	32 (5.1)	50 (4.0)	
	Same neighborhood, town/city, or province	497 (79.5)	454 (72.6)	951 (76.1)	
	Other province or country	110 (17.6)	139 (22.2)	249 (19.9)	
Type of relationship	Direct relative	331 (53.0)	346 (55.4)	677 (54.2)	*p* = 0.523
	Friend, neighbor, or work colleague	240 (38.4)	230 (36.8)	470 (37.6)	
	Health care professional or social worker	24 (3.8)	21 (3.4)	45 (3.6)	
	Non-professional offering paid help	8 (1.3)	3 (0.5)	11 (0.9)	
	Other	22 (3.5)	24 (4.0)	47 (3.8)	
Proximity	Close	517 (82.7)	499 (79.8)	1016 (81.3)	*p* = 0.192
	Not close	108 (17.3)	126 (20.2)	234 (18.7)	

* Significance: *p* < 0.05.

**Table 3:** Structural characteristics of the personal networks of male and female caregivers.

	Minimum		Maximum		Mean			Standard Deviation	
	M	F	M	F	M	F	*p*-Value	M	F
	(*n* = 25)	(*n* = 25)	(*n* = 25)	(*n* = 25)	(*n* = 25)	(*n* = 25)		(*n* = 25)	(*n* = 25)
Density	0.307	0.330	1	0.997	0.62756	0.59232	0.500	0.211688	0.149511
Degree_Mean	7.36	7.92	24.00	23.92	15.06	14.21	0.500	5.08	3.59
Betweenness_Mean	0.00	0.04	12.92	8.40	4.94	4.90	0.961	3.32	1.76
No. of cliques	1.00	2.00	33.00	21.00	11.92	11.08	0.667	8.42	4.86
No. of components	1.00	1.00	2.00	2.00	1.04	1.04	1	0.20	0.20
No. of isolates	0.00	0.00	1.00	1.00	0.08	0.12	0.646	0.28	0.33

Abbreviations: F, female; M, male; T, test for independent samples; significance, *p* < 0.05.

**Table 4:** Type of support received by male and female caregivers.

Type of Support		Male Egos	Female Egos	All Egos	*p*-Value
		*n* = 625 Alters	*n* = 625 Alters	*n* = 1250 Alters	
		*n* (%)	*n* (%)	*n* (%)	
Emotional support	No	249 (39.8)	238 (38.1)	487 (39.0)	*p* = 0.012
	Yes, for caregiving-related matters	31 (5.0)	57 (9.1)	88 (7.0)	
	Yes, for non-caregiving-related matters	35 (5.6)	22 (3.5)	57 (4.6)	
	Yes, for both of the above situations	310 (49.6)	308 (49.3)	618 (49.4)	
Financial support	No	583 (93.3)	581 (93.0)	1164 (93.1)	*p* = 0.102
	Yes, for caregiving-related matters	6 (1.0)	7 (1.1)	13 (1.0)	
	Yes, for non-caregiving-related matters	14 (2.2)	5 (0.8)	19 (1.5)	
	Yes, for both of the above situations	22 (3.5)	32 (5.1)	54 (4.3)	
		**Male Egos**	**Female Egos**	**All Egos**	***p*-Value**
		***n* Alter (%)**	***n* Alter (%)**	***n* Alter (%)**	
Help with specific caregiving tasks *	Personal care				0.635
	Yes	74 (52.5)	68 (49.6)	142 (51.1)	
	No	67(47.5)	69 (50.4)	136 (48.9)	
	Household chores				0.075
	Yes	67 (45.6)	49 (35.3)	116 (40.6)	
	No	80 (54.4)	90 (64.7)	160 (59.4)	
	Nursing-type tasks				0.713
	Yes	74 (50.3)	73 (52.5)	147 (51.4)	
	No	73 (49.7)	66 (47.5)	139 (48.6)	
	Supervising and keeping dependent person company				0.981
	Yes	95 (64.6)	89 (64.5)	184 (64.6)	
	No	52 (35.4)	49 (35.5)	101 (35.4)	
	Physical mobility				0.269
	Yes	84 (60.4)	87 (66.9)	171 (63.6)	
	No	55 (39.6)	43 (33.1)	98 (36.4)	
	Caregiving tasks outside the home				0.344
	Yes	92 (63.0)	95 (68.3)	187 (65.6)	
	No	54 (37.0)	44 (31.7)	98 (34.4)	

* N includes those that help to ego when that task is required.

**Table 5:** The odds of poor self-perceived health among caregivers according to number of direct relatives in personal network, reception of caregiving support, and mean degree centrality (bivariate analyses, ORs adjusted for age; 95% CI).

Variables		Total Egos	
		*n* = 50	
		OR for Poor Self-Perceived Health	(95% CI)
Gender	Male	1	
	Female	1.026	(0.307–3.432)
Composition variable	Presence of direct relatives in personal networks	0.979	(0.946–1.013)
Social support function variable	Presence of people in network offering help with specific caregiving tasks	1.015	(0.972–1.058)
Structural variable	Degree centrality mean	0.935	(0.254–3.451)

Abbreviations: CI, confidence interval; OR, odds ratio.

## Appendix A

English translation of original Spanish questionnaire used in the interviews.

Questions	Answer Categories
EGO MODULE	
Caregiver (ego) ID code	
Are you a man or a woman?	Man
	Woman
How old are you?	
ALTER PROMPT MODULE	
Name generator question. Please tell me the names of 25 people who form part of your life, whom you know by name and vice versa, and with whom you have had contact in the last 2 years.	
ALTER MODULE	
Is this person a man or woman?	Man
	Woman
How old is this person?	
What is this person’s relationship with you?	Partner
	Child
	Parent
	Brother/sister
	Another type of relative
	Friend
	Neighbor
	Work colleague
	Health care professional or social worker
	Non-professional offering paid help
	Other
Where does this person live with respect to you?	Same home
	Same neighborhood
	Same village/town/city
	Same province
	Other province
	Other country
How close do you feel to this person?	Very close
	Quite close
	Close
	Not very close
	Not at all close
Does this person help you financially?	No
	Yes, for caregiving-related matters
	Yes, for non-caregiving-related matters
	Yes, for both of the above situations
Does this person offer you emotional support?	No
	Yes, for caregiving-related matters
	Yes, for non-caregiving-related matters
	Yes, for both of the above situations
Help with caregivingDo you receive help with caregiving (specific caregiving tasks) from this person?	Yes
	No (none)
	It’s the person I care for
	He/she temporarily replaces me (caregiving shifts)
Do you receive help with personal care tasks from this person?	The person I care for DOES NOT NEED this type of care
	I do NOT receive help with personal care tasks from this person (the person I care for DOES need this type of care)
	YES I receive help with tasks of this kind from this person
Do you receive help with physical mobility tasks from this person?	The person I care for DOES NOT NEED this type of care
	I do NOT receive help with personal care tasks from this person (the person I care for DOES need this type of care)
	YES I receive help with tasks of this kind from this person
Do you receive help with household chores from this person?	The person I care for DOES NOT NEED this type of care
	I do NOT receive help with personal care tasks from this person (the person I care for DOES need this type of care)
	YES I receive help with tasks of this kind from this person
Do you receive help with supervising or keeping the person in your care company at home from this person?	The person I care for DOES NOT NEED this type of care
	I do NOT receive help with personal care tasks from this person (the person I care for DOES need this type of care)
	YES I receive help with tasks of this kind from this person
Do you receive help with nursing-type tasks from this person?	The person I care for DOES NOT NEED this type of care
	I do NOT receive help with personal care tasks from this person (the person I care for DOES need this type of care)
	YES I receive help with tasks of this kind from this person
Do you receive help with caregiving tasks outside the home from this person?	The person I care for DOES NOT NEED this type of care
	I do NOT receive help with personal care tasks from this person (the person I care for DOES need this type of care)
	YES I receive help with tasks of this kind from this person
How often do you receive support from this person…	Every day
	Two or three times a week
	Every week
	Every 2 weeks
	Every month
	Every 2 or 3 months
	Every 4 months or more
Have you helped this person?	Yes
	No
In the past year, has the caregiving support this person offers you…	Increased
	Decreased
	Remained the same
ALTER PAIR MODULE	
Do you think that these two people have an actual relationship?	It is quite likely
	Yes
	No
